# Supplementary material for: Mucus-Penetrating Silk Fibroin-Based Nanotherapeutics for Efficient Treatment of Ulcerative Colitis
Source: Biomolecules. 2022 Sep 8;12(9):1263. doi: 10.3390/biom12091263 (PMC9496219; doi:10.3390/biom12091263)
Supplement: Supplementary file 1 [file biomolecules-12-01263-s001.zip › biomolecules-1868768-supplementary.pdf]

Supplementary Materials

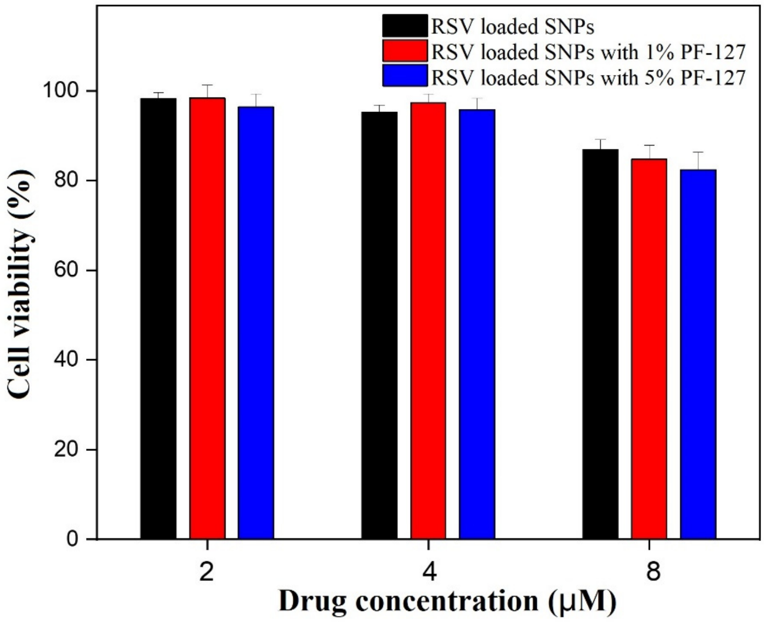

Figure S1. Cell viability of macrophages after incubation with various SNPs for 24 h. Data are expressed as means ± S.E.M. (n = 5).

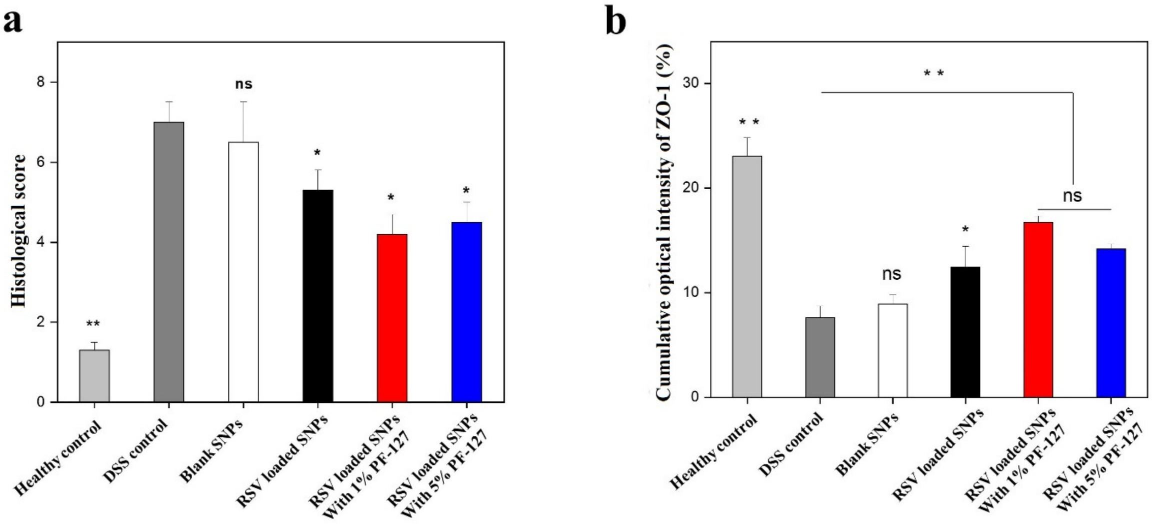

Figure S2. (a) Histological scores from H&E stained colons and (b) immunohistochemical analysis of the expression profile of ZO-1. Each point represents the mean ± S.E.M. (n = 3).
